# The Prevalence of Sexual Violence among Female Refugees in Complex Humanitarian Emergencies: a Systematic Review and Meta-analysis

**DOI:** 10.1371/currents.dis.835f10778fd80ae031aac12d3b533ca7

**Published:** 2014-03-18

**Authors:** Alexander Vu, Atif Adam, Andrea Wirtz, Kiemanh Pham, Leonard Rubenstein, Nancy Glass, Chris Beyrer, Sonal Singh

**Affiliations:** International Emergency Medicine and Public Health Fellowship Program, Department of Emergency Medicine, Johns Hopkins School of Medicine; Health Systems Program, Department of International Health, The Johns Hopkins Bloomberg School of Public Health, Baltimore, Maryland, USA; Department of International Health, Johns Hopkins Bloomberg School of Public Health, Baltimore, Maryland, USA; Department of Emergency Medicine, Johns Hopkins School of Medicine; Center for Public Health and Human Rights, Department of Epidemiology, Johns Hopkins Bloomberg School of Public Health, Baltimore, Maryland, USA; International Emergency Medicine and Public Health Fellowship Program, Department of Emergency Medicine, Johns Hopkins School of Medicine, Baltimore, Maryland, USA; Center for Public Health and Human Rights, Department of Epidemiology, Johns Hopkins Bloomberg School of Public Health, Baltimore, Maryland, USA; Johns Hopkins University School of Nursing, Department of International Health, Johns Hopkins Bloomberg School of Public Health, Baltimore, Maryland, USA; Center for Public Health and Human Rights, Department of Epidemiology, Johns Hopkins Bloomberg School of Public Health, Baltimore, Maryland, USA; Department of Medicine, Johns Hopkins School of Medicine; Center for Public Health and Human Rights, Department of Epidemiology, Johns Hopkins Bloomberg School of Public Health, Baltimore, Maryland, USA

## Abstract

Importance: Refugees and internally displaced persons are highly vulnerable to sexual violence during conflict and subsequent displacement. However, accurate estimates of the prevalence of sexual violence among in these populations remain uncertain. Objective: Our objective was to estimate the prevalence of sexual violence among refugees and displaced persons in complex humanitarian emergencies. Data Source: We conducted systematic review of relevant literature in multiple databases (EMBASE, CINAHL, and MEDLINE) through February 2013 to identify studies. We also reviewed reference lists of included articles to identify any missing sources. Study Selection: Inclusion criteria required identification of sexual violence among refugees and internally displaced persons or those displaced by conflict in complex humanitarian settings. Studies were excluded if they did not provide female sexual violence prevalence, or that included only single case reports, anecdotes, and those that focused on displacement associated with natural disasters. After a review of 1175 citations 19 unique studies were selected. Data Extraction: Two reviewers worked independently to identify final selection and a third reviewer adjudicated any differences. Descriptive and quantitative information was extracted; prevalence estimates were synthesized. Heterogeneity was assessed using I2. Main Outcomes: The main outcome of interest was sexual violence among female refugees and internally displaced persons in complex humanitarian settings. Results: The prevalence of sexual violence was estimated at 21.4% (95% CI, 14.9-28.7; I2=98.3%), using a random effects model. Statistical heterogeneity was noted with studies using probability sampling designs reporting lower prevalence of sexual violence (21.0%, 95% CI, 13.2-30.1; I2=98.6%), compared to lower quality studies (21.7%, 95% CI, 11.5-34.2; I2=97.4%). We could not rule out the presence of publication bias. Conclusions: The findings suggest that approximately one in five refugees or displaced women in complex humanitarian settings experienced sexual violence. However, this is likely an underestimation of the true prevalence given the multiple existing barriers associated with disclosure. The long-term health and social consequences of sexual violence for women and their families necessitate strategies to improve identification of survivors of sexual violence and increase prevention and response interventions in these complex settings.

## Background

War places civilians at increased risk of many forms of violence. The fear of violence can promote forced and mass displacement. As of January 2012, almost 11 million people were registered as refugees or internally displaced persons (IDPs), as estimated by UNHCR.^1^ During transitions through conflict and displacement, refugees and IDPs continue to live at heightened vulnerability to violence due to breakdown of family and social structure, and changes to law enforcement and protective structures.^2^^,^ 3 ^,^ 4 Significant efforts have been made to assess for, prevent and respond to gender-based violence (GBV) that occurs in these settings.^2^^,^ 3 ^,^ 4 ^,^ 5 However, GBV is broadly defined.^6^ For the purpose of this study, we focus on sexual violence as defined by the US Center for Disease Control and Prevention^7^ :

…any nonconsensual completed or attempted contact (between the penis and the vulva or the penis and the anus involving penetration, however slight), nonconsensual contact between the mouth and the penis, vulva, or anus; nonconsensual penetration of the anal or genital opening of another person by a hand, finger, or other object; nonconsensual intentional touching, either directly or through the clothing, of the genitalia, anus, groin, breast, inner thigh, or buttocks; or nonconsensual non-contact acts of a sexual nature such as voyeurism and verbal or behavioral sexual harassment. All the above acts also qualify as sexual violence if they are committed against someone who is unable to consent or refuse…

Displaced women and girls are vulnerable to a range of sexual violence including forced sex/rape, sexual abuse by an intimate partner, child sexual abuse, coerced sex, and sex trafficking in conflict and humanitarian settings.^8^ Many studies have focused on the issue of rape as a weapon of war, leading to assumptions that armed actors and military personnel are the main perpetrators of sexual violence.^9^ Other perpetrators, however, may also include family members, NGO and humanitarian workers, trusted individuals, or strangers who take advantage of heightened vulnerability.^10^^,^ 11 As a result, women and girls who experience sexual violence may experience a range of long lasting physical,^12^^,^ 13 reproductive,^14^^,^ 15 ^,^ 16 and mental health consequences of sexual violence.^13^

Prevention and response to sexual violence in humanitarian settings focuses on three main areas of medical and reproductive care, psychosocial support, and protection.^5^^,^ 17 ^,^ 18 ^,^ 19 ^,^ 20 ^,^ 21 ^,^ 22 ^,^ 23 To ensure quality and coverage of these services, however, donors and humanitarian organizations must make evidence-informed decisions with inputs related to level of need, intervention costs, and other priority needs of the displaced population.^24^ To this end, response efforts for sexual violence have been hampered by the lack of an adequate epidemiological understanding of the true estimates of sexual violence among refugees and IDPs. The available literature is scant and unreliable.^25^ The little research that is available has reported varying prevalence estimates, some with extremely high or low figures,^26^ leading to concerns about inappropriate estimations of both the true magnitude of sexual violence and the contexts in which sexual violence occurs.^10^^,^ 27 Differences in sampling techniques, definitions and recall periods of sexual violence, ethical considerations, or the challenging nature of conducting research in complex humanitarian emergencies may partially explain such differences in estimates.^27^

Though there have been systematic reviews of gender-based violence^26^ and sexual violence,^9^ to our knowledge, there has been no published meta-analysis of prevalence of sexual violence among displaced populations that has attempted to combine and contrast the estimates from across these different studies. Our objective is to estimate the prevalence of sexual violence among refugees and internally displaced persons in complex humanitarian emergencies.

## Methods

**Literature Search**

The meta-analysis was conducted according to a pre-specified protocol available from the investigators. We conducted an initial search on MEDLINE, EMBASE and CINAHL in November 2010 using an enhanced filter in consultation with an information specialist. Details of our search strategy and terms are presented in the Online Supplement (**Appendix 1**). The search was optimized for sensitivity and specificity through key articles identified by experts. At the time of manuscript preparation, we updated the search on February 2013 and also evaluated the bibliographies of included studies for relevant publications.

We restricted our searches to studies published in English and developed a search strategy for MEDLINE based on medical subject headings (MeSH) terms and text words of key articles that we identified a priori. Studies were not included or excluded on the basis of design but were required to report (or have references) on their study design and methodologies utilized. Inclusion criteria required the description of an evaluation of a screening tool, strategy, survey, or program to identify sexual violence among refugees and IDPs or those displaced by conflict in complex humanitarian settings. We excluded studies that did not provide prevalence of sexual violence for females, or that included only single case reports, anecdotes, and those that focused on displacement associated with natural disasters. We excluded studies that focused on female genital mutilation as a form of sexual violence. We excluded studies in which it was unclear as to whether the study population was migrant or refugees or if the results were not stratified on the basis of migrant or refugee/IDP status. Two reviewers worked independently and in duplicate, to review titles, abstracts, and full text versions of identified reports. A third reviewer met to discuss and to adjudicate differences.

**Data Abstraction**

Following the search, duplicate publications were removed. Preliminary screening included a review of all titles and abstracts of identified studies from our searches, excluding those that failed to meet selection criteria. The remaining articles underwent full-text evaluation for inclusion eligibility. Data were abstracted from identified studies that reported the outcome measures of female-targeted sexual violence, prevalence estimates, and other associations.

**Study Characteristic*s***

We extracted information related to the study characteristics, including country where the study was conducted, country of origin of study population, participant age range, proportion of female participants within the study sample, and total sample size of female participants. We also extracted information that described the study design, including design sampling method, whether the target sample size was reached according to the study authors, non-response percentage. Characteristics of the instrument used to assess sexual violence were also collected, including if pilot testing of questionnaires was performed prior to data collection, the type of instrument(s) used, and validation measures (i.e. reporting of internal consistency, sensitivity, and specificity).

**Risk of bias**

In order to determine the risk of bias of studies, we evaluated whether reliability had been assessed and whether authors evaluated construct validity of the screening instruments. We also determined whether sampling was convenience or probability-based. We did not conduct quantitative tests for publication bias but assessed this qualitatively when relevant.

**Outcome Measure**

Sexual violence outcomes included reported rape, molestation, sexual abuse, genital mutilation, gang rape, marital rape, sexual violence related to exploitation, and sexual harassment, as reported by the authors. Though recognizing the importance of GBV on the health and well-being of displaced persons, we focused the search on sexual violence among female refugees and IDPs, as opposed to GBV, given the broad definition,^6^ interpretation, and variable measurement of GBV in these settings.^2^

**Data Synthesis**

Statistical analysis was conducted using StatsDirect (version 2.7.9). Statistical heterogeneity was tested and prevalence proportions were pooled using a fixed-effect model if heterogeneity was limited; a random-effects model was used when there was a significant heterogeneity among the studies.^28^ Since heterogeneity was anticipated in these studies we considered the more conservative random-effects model as a more reliable estimate of the prevalence. To maintain similarity of sampling designs across the studies, we conducted sensitivity analyses to determine the robustness of the effect size when studies that had a defined sampling design were analyzed separately from those studies that reported non-probability based sampling methods. A conventional level of p<0.05 was utilized to assess significance. Our study was reported out according to the MOOSE Reporting instrument.^29^ The protocol is available on request from the corresponding author.

## Results

**Search results**

After a review of 1175 citations, we selected 19 unique studies that reported on prevalence of sexual violence among female refugees and IDPs in the setting of complex humanitarian emergencies.^3^^,^ 4 ^,^ 30 ^,^ 31 ^,^ 32 ^,^ 33 ^,^ 34 ^,^ 35 ^,^ 36 ^,^ 37 ^,^ 38 ^,^ 39 ^,^ 40 ^,^ 41 ^,^ 42 ^,^ 43 ^,^ 44 ^,^ 45 ^,^ 46 The selection of studies included in our review is summarized in **Figure 1**.

**Selection Algorithm:**
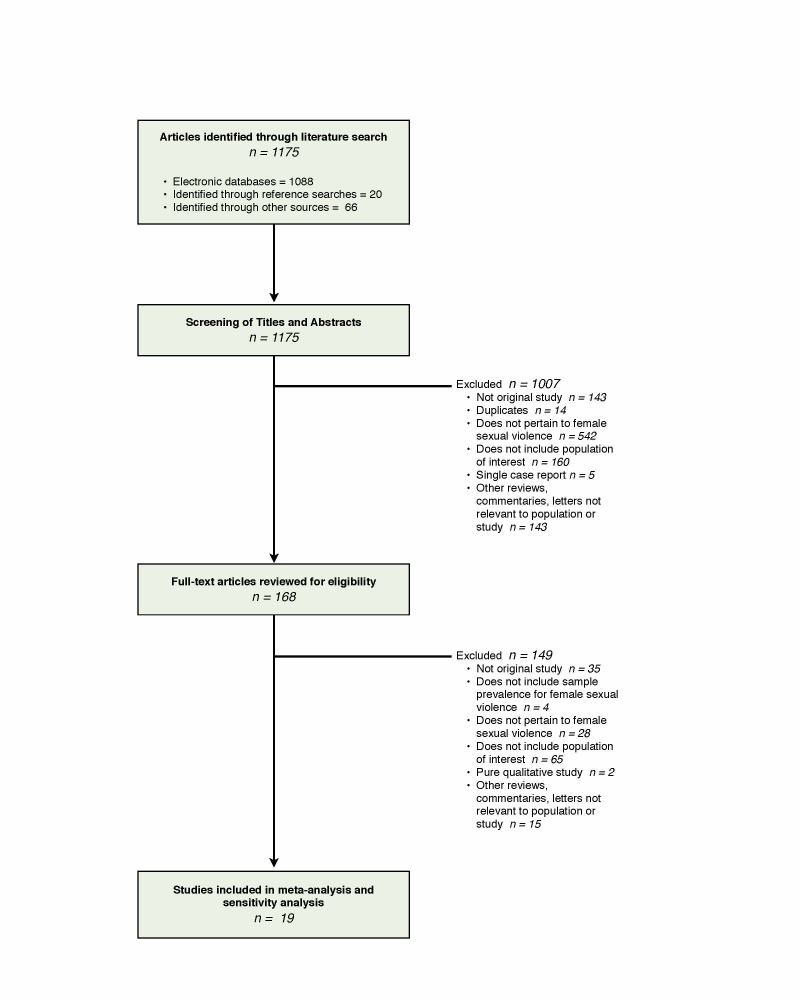

**Study characteristics**

Characteristics of studies are summarized in Table 1. The 19 studies enrolled a total of 8398 participants. Participants of the studies were either refugees or IDPs from 14 different countries of origin that were affected by conflict and 14 different countries where the studies were conducted. The proportion of female participants ranged from 26.5% to 100%. The study sample sizes ranged from 34 to 991. There was significant variability of the age range; the widest reported age range was 11-70 years of age.

**Table 1: Study characteristics:** 

**Study Author (Year of study)**	**Country of Study**	**Country of Origin of refugee/displaced**	**Age Range (years)**	**Proportion of Female ** **Participants (%)**	**No. of Females in Sample (N)**	
**McKelvey (1995)**	Philippines	Vietnam	18.0 - 25.0	33.0	34	
**Swiss (1998)**	Liberia	Liberia	Not reported	100.0	205	
**Petersen (2000)**	Thailand	Burma/Myanmar	11.0 - 70.0	37.0	48	
**Cardozo (2000)**	Kosovo	Kosovo	Not reported	62.3	825	
**Amowitz (2002)**	Sierra Leone	Sierra Leone	14.0 - 80.0	100.0	991	
**Kerimova (2003)**	Azerbaijan	Azerbaijan	33.5 *	100.0	457	
**Hynes (2004)**	E. Timor	E. Timor	18.0 - 49.0	100.0	287	
**Avdibegović (2006)**	Bosnia and Herzegovina	Bosnia and Herzegovina	43.0 *	100.0	283	
**Amone-P'Olak (2006) **	Uganda	Uganda	12.0 - 19.0	26.5	78	
**Hammoury (2007)**	Lebanon	Palestine	28.0 *	100.0	349	
**Roberts (2008)**	Uganda	Uganda	35.3 *	60.1	727	
**Johnson (2008)**	Liberia	Liberia	40.2 - 42.4	52.8	880	
**Usta (2008)**	Lebanon	Lebanon	15.0 - 72.0	100.0	310	
**Hagan (2009)**	Chad	Sudan	37.1 *	60.0	559	
**Kinyanda (2010)**	Uganda	Uganda	24.0 *	70.5	573	
**Vinck (2010)**	Central African Republic	Central African Republic	36.4 *	49.8	936	
**Johnson (2010)**	DRC	DRC	38.2 - 42.0	59.4	586	
**Betancourt (2011)**	Sierra Leon	Sierra Leon	16.2 *	28.9	79	
**Parmar (2012)**	Eastern Cameroon	Central African Republic	35.1 *	100.0	191	
* Age range not available. Mean age reported instead						

**Design and risk of bias results**

**Table 2** summarizes the study designs and the survey instruments used to assess sexual violence among female refugees and IDPs, as reported by the study authors. Among the nineteen selected studies, 11 studies utilized probability based random sampling methods^3^^,^ 4 ^,^ 30 ^,^ 34 ^,^ 35 ^,^ 36 ^,^ 39 ^,^ 41 ^,^ 43 ^,^ 44 ^,^ 46 and eight utilized non-probability based sampling methods.^31^^,^ 32 ^,^ 33 ^,^ 37 ^,^ 38 ^,^ 40 ^,^ 42 ^,^ 45 Definitions of sexual violence ranged from “improper sexual acts of any kind”^45^ to narrowly specified acts of sexual violence such as coerced penetration.^4^^,^ 34 ^,^ 36 Recall periods of sexual violence varied from 6 months^41^ to lifetime.^4^^,^ 31 ^,^ 34 ^,^ 39 Many of the studies (57.9%) did not develop the sample size with the appropriate effect size to measure the prevalence of sexual violence as the principal aim of the study. For example, several studies used a subsample of a larger study population to estimate the sexual violence prevalence.^30^^,^ 31 ^,^ 32 ^,^ 33 ^,^ 39 ^,^ 40 ^,^ 42 ^,^ 43 ^,^ 44 ^,^ 45 ^,^ 46 Six studies reported that they had reached the targeted sample size.^33^^,^ 35 ^,^ 39 ^,^ 41 ^,^ 43 ^,^ 46 Another three studies reported that targeted sample size was not reached.^4^^,^ 34 ^,^ 36 Non-response rate was reported in nine studies.^4^^,^ 31 ^,^ 33 ^,^ 34 ^,^ 35 ^,^ 36 ^,^ 40 ^,^ 41 ^,^ 46 Five studies had reported that pilot testing of the survey instruments was carried out prior to data collection.^4^^,^ 33 ^,^ 34 ^,^ 36 ^,^ 46

**Table 2: Reported Study Design and Study Instrument Used to Assess Sexual Violence:** 

**Study Author (Year of study)**	**Study Design**				**SV Assessment Instrument**	
	**Probability vs. Non-Probability**	**Sample Size Adequacy Reached**	**Non- response**	**Pilot Testing**	**Type of Instrument Used**	**Sensitivity/ Specificity**
**McKelvey (1995)**	Non-Probability	Not reported	5.9%	Not reported	Survey/Questionnaire	Not reported
**Swiss (1998)**	Probability	Not reported	Not reported	No	Survey/Questionnaire	Not reported
**Petersen (2000)**	Non-Probability	Not reported	Not reported	Not reported	Survey/Questionnaire	Not reported
**Cardozo (2000)**	Probability	Yes	Not reported	No	HTQ	Not reported
**Amowitz (2002)**	Probability	No	5.0%	Yes	Survey/Questionnaire	Not reported
**Kerimova (2003)**	Non-Probability	Not reported	Not reported	No	Survey/Questionnaire	Not reported
**Hynes (2004)**	Probability	No	26.0%	Yes	Survey/Questionnaire	Not reported
**Avdibegović (2006)**	Non-Probability	Not reported	0.0%	Not reported	Survey/Questionnaire	Not reported
**Amone-P'Olak (2006) **	Probability	Not reported	Not reported	Not reported	Survey/Questionnaire	Not reported
**Hammoury (2007)**	Non-Probability	Yes	0.6%	Yes	AAS	Not reported
**Roberts (2008)**	Probability	Yes	Not reported	Not reported	HTQ	Not reported
**Johnson (2008)**	Probability	Yes	1.8%	Not reported	Survey/Questionnaire	Not reported
**Usta (2008)**	Non-Probability	Not reported	Not reported	No	Survey/Questionnaire	Not reported
**Hagan (2009)**	Probability	Not reported	Not reported	No	Survey/Questionnaire	Not reported
**Kinyanda (2010)**	Non-Probability	Not reported	Not reported	No	Survey/Questionnaire	Not reported
**Vinck (2010)**	Probability	Yes	5.0%	Yes	Survey/Questionnaire	Not reported
**Johnson (2010)**	Probability	No	1.1%	Yes	Survey/Questionnaire	Not reported
**Betancourt (2011)**	Non-Probability	Not reported	Not reported	No	Survey/Questionnaire	Not reported
**Parmar (2012)**	Probability	Yes	0.5%	No	Survey/Questionnaire	Not reported
*HTQ- Harvard Trauma Questionnaire **AAS- Abuse Assessment Screen****						

The survey instruments used to assess for sexual violence included the Harvard Trauma Questionnaire (HTQ),^47^^,^ 48 , the Abuse Assessment Screen (AAS)^49^ and survey questionnaires (validation of survey questionnaires were not reported). Internal consistency (Cronbach’s α) of the instruments used was only reported in the Roberts study^43^ that used HTQ. None of the studies reported sensitivity and specificity of the instrument used to assess for sexual violence. We could not rule out the presence of publication bias among the included studies.

**Prevalence proportion for sexual violence**

The estimated prevalence of sexual violence among the 19 selected studies^3^^,^ 4 ^,^ 30 ^,^ 31 ^,^ 32 ^,^ 33 ^,^ 34 ^,^ 35 ^,^ 36 ^,^ 37 ^,^ 38 ^,^ 39 ^,^ 40 ^,^ 41 ^,^ 42 ^,^ 43 ^,^ 44 ^,^ 45 ^,^ 46 was 21.4% (1521/8398; 95% CI, 14.9-28.7; I^2^=98.3), using the random effects statistical model (**Figure 2**). A sensitivity analysis was performed to compare the prevalence proportion of sexual violence of the studies that used probability based random sampling to the studies that used non-probability based sampling (**Appendix 2**). Compared to the primary analysis, the 11 studies that utilized probability based sampling methodologies noted an estimated 21.0% prevalence of sexual violence (961/6265; 95% CI, 13.2-30.1; I^2^=98.6%) using the random-effects model. The eight studies that used non-probability based sampling methodologies produced an estimated prevalence of sexual violence of 21.7% (560/2133; 95% CI, 11.5-34.2; I^2^=97.4%) using the random-effects model. Another sensitivity analysis was done to compare studies that focused on sexual violence as the primary objective compared to studies that was not dedicated to sexual violence as the primary objective (**Appendix 2**). Compared to the primary analysis, the 9 studies that focused on sexual violence as the primary objective yielded in an estimated 20.7% (911/4476; 95% CI, 13.0-29.6; I^2^= 97.8%) compared to the 10 studies with other primary objectives with an estimated 22.2% (610/3922; 95% CI, 11.8-34.7; I^2^= 98.6%) prevalence of sexual violence using random effects model.

**Meta-analysis plot:**
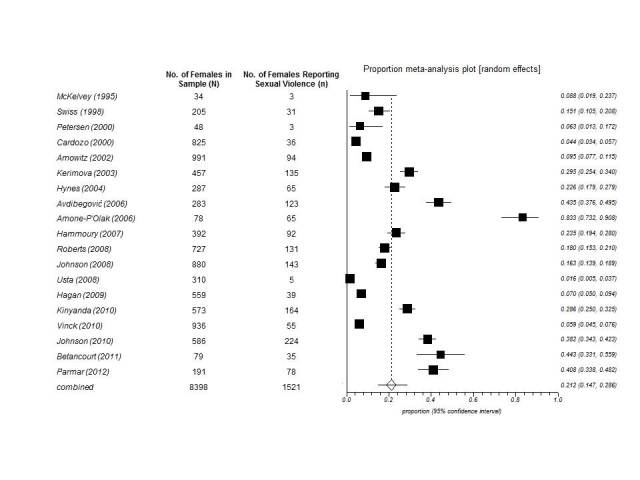

## Discussion

In the present meta-analysis of 19 studies, we found the prevalence of sexual violence among female refugees and internally displaced persons across 14 countries affected by conflict to be 21.4%. The quantification of sexual violence among female refugees and IDPs in complex humanitarian emergencies is challenging.

Sexual violence is often under-reported. The social stigma associated with rape, shame and fear of reprisal are significant deterrents for survivors of sexual violence to report their traumatic experiences. Compounding social stigma is an often inadequate justice system response that fails to arrest or prosecute perpetrators, a law enforcement system that often mistreats and further victimizes survivors of sexual violence, and a lack of capacity of service providers across multiple systems to receive and give adequate attention to the various and complex needs of women who have been raped.^2^ The cumulative effect is an inhospitable climate for survivors to come forth to disclose their experience and to seek help. The negative health impacts of the experience of sexual violence are significant and long-term and may, include serious physical injuries, sexually transmitted infections and HIV infections,^16^^,^ 50 fistulas and chronic pain,^51^^,^ 52 unwanted pregnancies,^53^ and a myriad of psychological health consequences including suicide.^43^^,^ 45 ^,^ 54 ^,^ 55

The body of research aimed at understanding the prevalence of sexual violence among refugees and displaced populations in conflicts is exponentially more difficult to elucidate given the context and the sensitive subject matter. Not only is there difficulty in reaching the affected populations, but investigators also employ varying definitions and research methodologies to estimate prevalence of sexual violence. Even when taking into account the methodological issues, our finding provides evidence of the need for concerted action to address sexual violence among refugees and displaced populations in conflict settings. The estimated lifetime prevalence of sexual violence against women in non-conflict settings ranges from 10%-50% depending on the country and who is identified as the perpetrator (e.g. intimate partner vs. stranger).^13^^,^ 56 In many countries where these estimates are collected in non-conflict setting, basic justice and law enforcement systems are in place. Situations of conflict and displacement may exacerbate existing gender based violence in families and communities and present new forms of violence (e.g. sexual slavery) against women and girls.^53^ Hence, the prevalence of sexual violence of 21.4% among refugees and IDPs still likely underestimates the true prevalence as many incidences of sexual violence in the setting of complex humanitarian emergencies, such as conflict, go unreported.

Similar to the recent systematic review by Stark and Ager,^26^ which focused on the prevalence of GBV in complex emergencies, the systematic review suggests a need for more uniform methods and common definitions in future research. Greater uniformity would yield deeper understanding of sexual violence among refugee and displaced populations. Adopting more consistent approaches, moreover, could potentially identify greater incidence of survivors of sexual violence.

In addition to the importance of determining prevalence, there is also a critical need to understand who the range of perpetrators may be, as well as the physical locations and settings in which sexual violence is likely to occur. More vigorous action is needed to prevent and to respond to sexual violence among refugees and displaced populations, identify methods to assist survivors, and hold perpetrators accountable. Potential steps include strengthening procedures for identifying survivors, such as the integration of routine GBV screening inquiry for women and girls in protection and health programs using a validated measure with trained providers; expanding prevention efforts using social norms perspectives, providing response services such as psychosocial services and livelihood programs for survivors;^23^ building competency, compassion and collaboration among protection officers, health and other service providers, and police; and providing resources and political will to investigate and prosecute perpetrators.****

## Limitations

Our analysis has limitations. Our search focused on peer-reviewed publications and searches of secondary sources. Our search may have exclude non-English articles and non peer-reviewed reports by humanitarian organizations. Although sexual violence is a global phenomenon, the search identified studies of refugees or displaced persons from predominantly African countries and thus limits the generalizability of our findings to other contexts. The results may be influenced by bias inherent in individual studies, particularly including social desirability bias that often accompanies self-reported responses to sensitive questions.^57^ Some studies evaluated violence and traumatic events that occurred in very distant past or did not report the time of the event, thus it is important to note that we cannot deduce estimates of temporality. Since there are no uniformly accepted measures of risk of bias assessments of cross-sectional studies, we developed our own assessment scale for this context. Additionally, some of the select 19 studies included in the final quantitative synthesis were not dedicated to assessing sexual violence but rather on other related topics in which sexual violence questions were included in the survey. We have performed a sensitivity analysis to compare the studies with the primary objective to measure sexual violence compared to studies with other primary objectives and we found no significant difference. The ideal study focused on assessing prevalence of sexual violence should include survey questions that are based on specific types and acts of sexual violence and delivered by trained interviewers in private confidential settings. As some of the studies were not dedicated studies focused on sexual violence and many of the studies did not detail how the questionnaires were administered, it is unclear how the final prevalence proportions reported were affected.

While this analysis focused on sexual violence among females, sexual violence among males exists and warrants future attention. Emerging reports have documented sexual and gender-based violence among male refugee and IDP populations 23 ^,^ 35 ^,^ 58 ^,^ 59 and response to prevent and address health and social outcomes among these men have been emphasized as a critical component for an inclusive and comprehensive response to sexual and gender-based violence among displaced populations.^60^ We acknowledge this as an important area of study and believe a separate study to assess the prevalence of sexual violence against men in displaced setting is warranted to give due justice and attention to the issue.

## Conclusions

The findings suggest that approximately one in five refugees or displaced women in complex humanitarian settings experienced sexual violence. However, this is likely an underestimation of the true prevalence given the multiple existing barriers associated with disclosure. The long-term health and social consequences of sexual violence for women and their families necessitate strategies to improve identification of survivors of sexual violence and increase prevention and response interventions in these complex settings.

## Appendix 1

### Search Terms

**Table:**

**PUBMED/MEDLINE**	**EMBASE**	**CINAHL**
*Questionnaires; Tool; Evaluation; Measurement; Diagnosis; Diagnostic tool; Assessment*	*Questionnaires; Evaluation; Measurement; Screening *	
*Child Abuse; Dowry-related violence; Rape; Marital rape; Circumcision, female; Female genital mutilation; Female genital cutting; Non-spousal violence; Sexual violence related to exploitation; Sexual Harassment; Sexual harassment; Trafficking; Forced prostitution; Systematic rape; Sexual slavery*	Partner violence; Sexual abuse; Rape; Child sexual abuse; Prostitution	Intimate partner violence; Sexual abuse; Rape; Child sexual abuse;
*Refugees; Emigrants; Immigrants; Transients; War*	*Refugees; War; Conflict*	

## Appendix 2

### Sensitivity analysis for reported prevalence of sexual violence

**Table:**

**Types of studies included in sensitivity analysis**	**Statistical Model**	**No. of Studies**	**No. Events, n/N**	**Prevalence Proportion, % (95% CI)**
**Probability based random sampling**	Random	11	961/6265	21.0 (13.2-30.1)
**Non-probability based sampling**	Random	8	560/2133	21.7 (11.5-34.2)
**Studies with primary objective to measure sexual violence**	Random	9	911/4476	20.7 (13.0-29.6)
**Studies with other objective but reported on sexual violence**	Random	10	610/3922	22.2 (11.8-34.7)

## Appendix 3

### MOOSE Checklist

Download PDF

### PRISMA Checklist

Download PDF
